# Algebraic Study of diatomic Molecules: homonuclear molecules *H*_2_ and *N*_2_

**DOI:** 10.1038/s41598-020-64266-z

**Published:** 2020-05-06

**Authors:** N. Amiri, M. Ghapanvari, M. A. Jafarizadeh

**Affiliations:** 1 Department of Nuclear Physics, University of Tabriz, Tabriz, 51664 Iran; 2 Plasma Physics and Fusion Research School, Nuclear Science and Technology Research Institute, Tehran, Iran; 3 Department of Theoretical Physics and Astrophysics, University of Tabriz, Tabriz, 51664 Iran

**Keywords:** Chemistry, Mathematics and computing

## Abstract

It is the aim of this study to discuss for two-body systems like homonuclear molecules in which eigenvalues and eigenfunctions are obtained by exact solutions of the solvable models based on *SU*(1, 1) Lie algebras. Exact solutions of the solvable Hamiltonian regarding the relative motion in a two-body system on Lie algebras were obtained. The *U*(1) ↔ *O*(2), *U*(3) ↔ *O*(4) and *U*_*q*_(3) ↔ *O*_*q*_(4) transitional Hamiltonians are employed to described for *H*_2_ and *N*_2_ molecules. Applications to the rotation-vibration spectrum for the diatomic molecule indicate that complicated Hamiltonian can be easily determined via the exactly solvable method. The results confirm the mixing of both vibrating and rotating structures in *H*_2_ and *N*_2_ molecules.

## Introduction

The studies of molecular spectra of diatomic molecules are of great interest. Different ways are to the study of molecular spectra that require a large number of parameters to account for the structure of the molecules. Algebraic methods are one of the most useful methods for studying molecules. The main features and applications of Lie algebraic methods have been described in books^1,2^ and review articles^3^ in the last few years. There are many studies based on the interaction boson model(IBM)^4–6^. This Lie algebraic method is based on the second quantization of quantum numbers within the creation and annihilation operators.

**Figure 1:**
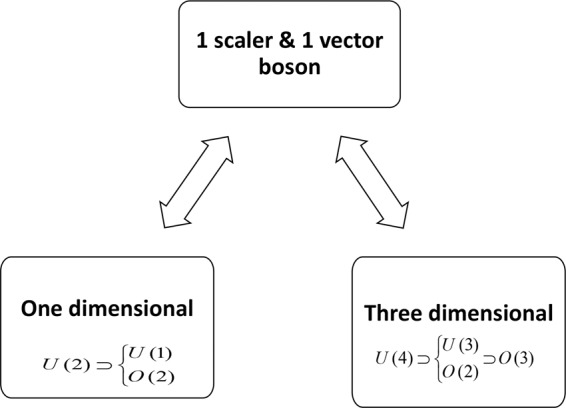
Various limiting cases of this theory.

The diatomic molecules are like two-body systems. Two-body systems have one-dimensional and three-dimensional algebraic models corresponding with algebra $U\mathrm{(2)}$ and algebra $U\mathrm{(4)}$, respectively (see Fig. 1). Various important features of the quantum algebraic formula for both the one and three-dimensional (exactly solvable) have been checked using suitable dynamical symmetry^7^.

The $U\mathrm{(4)}$ and $U\mathrm{(2)}$ algebraic models in the analysis of experimental data have been used so far in recent years. Rotations and vibrations are treated simultaneously in the $U\mathrm{(4)}$ model. The $U\mathrm{(2)}$ model treats rotations and vibrations separately. Iachello, Levine, and co-workers have described the rotation-vibration spectra of diatomic and triatomic molecules^8–12^ using $U\mathrm{(4)}$ algebra. In ref. ^13^, the experimental vibrational spectra of small and medium-sized molecules have been studied by algebraic techniques. These techniques are based on the idea of dynamical symmetry $U\mathrm{(2)}$ algebra.

In ref. ^14^, vibrational spectra in diatomic molecules in sentences of the $q$ deformed anharmonic oscillator based on the ${U}_{q}(2)\supset {O}_{q}(2)$ symmetry have been characterized. The different uses of quantum deformed algebraic have effected in nuclear and molecular physics^15,16^. The $q$-deformed IBM Hamiltonians were developed by Pan^17^, in which generators were used to construct the corresponding $q$-deformed Casimir operators. Then the $q$-deformed vibron model of the diatomic molecules is reported by Alvarez *et al*. in ref. ^18^. Also, we studied the phase transition of the even and odd nuclei based on $q$-deformed $SU\mathrm{(1,1)}$ algebraic model^19,20^.

In this paper, exact solutions of the solvable Hamiltonian about the relative motion in a two-body system on Lie algebras were obtained. One has to employ some complicated numerical methods to diagonalize the transitional Hamiltonian in analytic and exact solvable solutions of the duality paring models in diatomic molecules at rotational and vibrational modes, but Pan *et al*. in refs. ^21–23^ have suggested a new solution which is based on the affine $SU(1,1)$ Bethe ansatz algebraic technique. We have defined the molecular spectra for diatomic molecules by using transitional Hamiltonians which are based on the affine $SU(1,1)$ algebraic technique and quantum deformation theory^24–26^. We also considered the variation of the control parameter in the transitional theory. Our results propose a vibrational-rotational transition in the diatomic molecule and also explore the structures of the molecules. We have distinguished reasons to opt for the algebraic approach which constitutes a new method in the molecular system. The first reason is that it is a solvable model, a deformed version of the dynamical symmetries diatomic molecules has been constructed and we can have good accuracy in the study of energy spectra in the molecule.

The structure of this manuscript is as follows: section 2 briefly summarizes theoretical aspects of the transitional Hamiltonian, the affine $SU(1,1)$ algebraic technique and the $q$-deformed version for two-body systems. Section 3 includes the results and finally, Section 4 will contain discussion of the present results and plans for further work.

## Theoretical framework

These models are based on $U\mathrm{(2)}$ and $U\mathrm{(4)}$ lie algebra (Fig. 1). We start the discussion with a simple case of one-dimensional problems, described by the $U\mathrm{(2)}$ algebra.

### Transitional theory

The one-dimensional vibron model has been used for molecular spectroscopy. This algebra can be used to describe stretching vibrations of molecules. To provide a realization for the $U\mathrm{(2)}$ algebra we take two boson creation and annihilation operators, which we denote by ${s}^{\dagger }$, ${t}^{\dagger }$ and $s$, $t$. The $U\mathrm{(2)}$ algebra has four operators which can be realized as,

2.1 $${S}_{+}={t}^{\dagger }s,\,{S}_{-}={s}^{\dagger }t,\,{S}_{0}=\frac{1}{2}({t}^{\dagger }t-{s}^{\dagger }s),\,N={t}^{\dagger }t+{s}^{\dagger }s$$

The three operators ${S}_{+}$, ${S}_{-}$, ${S}_{0}$ are themselves closed under commutation and are elements of the algebra $SU\mathrm{(2)}$ which is a subalgebra of $U\mathrm{(2)}$. Using the commutations relationship given by,

2.2 $$[{S}_{+},{S}_{-}]=2{S}_{0},\,[{S}_{0},{S}_{\pm }]=\pm \,{S}_{\pm }$$

Dynamical symmetries for one-dimensional problems can be studied by considering all the possible subalgebras of $U\mathrm{(2)}$. The Casimir operator of $SU\mathrm{(1},\mathrm{1)}$ can be written as

23 $${\hat{C}}_{2}={S}^{0}({S}^{0}-1)-{S}^{+}{S}^{-}$$

The basis states of an irreducible representation $SU(1,1)$, $|k\mu \rangle$ are determined by a single number, $k$, where $k$ can be any positive number and $\mu =k,k+1,\ldots$. Therefore

24 $$\begin{array}{rcl}{S}^{\pm }\,|k\mu \rangle  & = & \sqrt{(\mu \pm k)(\mu \mp k\pm 1)}|k\mu \pm 1\rangle ,\\ {S}^{0}\,|k\mu \rangle  & = & \mu |k\mu \rangle ,\\ {\hat{C}}_{2}(SU(1,1))|k\mu \rangle  & = & k(k-1)|k\mu \rangle \end{array}$$

It is known that the bases of $U\mathrm{(1)}$ and $SO\mathrm{(2)}$ are simultaneously the basis of $S{U}^{t}(1,1)$ and $S{U}^{st}(1,1)$, respectively. Their complementary relations can be expressed as follows. We introduce $S{U}^{s}(1,1)$ and $S{U}^{t}(1,1)$ pairing algebras with

25 $$\begin{array}{rrl}{S}_{s}^{+}=\frac{1}{2}{s}^{\dagger }{s}^{\dagger }, & {S}_{s}^{-}=\frac{1}{2}\tilde{s}\tilde{s}, & {S}_{s}^{0}=\frac{1}{2}({s}^{\dagger }s+s{s}^{\dagger })=\frac{1}{2}{n}_{s}+\frac{1}{4}\\ {S}_{t}^{+}=\frac{1}{2}{t}^{\dagger }.{t}^{\dagger }, & {S}_{t}^{-}=\frac{1}{2}\tilde{t}.\tilde{t}, & {S}_{t}^{0}=\frac{1}{2}({t}^{\dagger }t+t{t}^{\dagger })=\frac{1}{2}{n}_{t}+\frac{3}{4}\end{array}$$

where ${n}_{s}$ and ${n}_{t}$ are the number operators for $s$ and $t$ bosons and $S{U}^{st}(1,1)$ is generated by the $s$ and $t$ boson pairing algebra. For the $U\mathrm{(1)}$ case one has

2.6 $$|N{n}_{t}\rangle =|N,{k}_{t}=\frac{1}{2}({n}_{t}+\frac{3}{2})\rangle $$

where $N$, ${n}_{t}$ and ${k}_{t}$ are quantum numbers of $U\mathrm{(2)}$, $U\mathrm{(1)}$ and $S{U}^{t}(1,1)$, respectively. The correspondence between the basis vectors of $SO\mathrm{(2)}$ and $S{U}^{st}(1,1)$ is

2.7 $$|N\nu \rangle =|N,{k}_{st}=\frac{1}{2}(\nu +2),{k}_{t}=\frac{1}{2}({n}_{t}+\frac{3}{2})\rangle $$

where $v$ is the $SO\mathrm{(2)}$ quantum number. Now, we introduce the operators of infinite dimensional algebra similar to what has been defined by Pan *et al*. in refs. ^21,22^,

28 $$\begin{array}{c}{S}_{n}^{\pm }={C}_{s}^{2n+1}{S}^{\pm }(s)+{C}_{t}^{2n+1}{S}^{\pm }(t)\\ {S}_{n}^{0}={C}_{s}^{2n}{S}^{0}(s)+{C}_{t}^{2n}{S}^{0}(t)\end{array}$$

where ${C}_{s}$ and ${C}_{t}$ are real parameters and $n$ can be taken $0,\pm \,1,\pm \,2,\ldots$. To evaluate the energy spectra and transition probabilities, let us consider $|lw\rangle$ as the lowest weight state of $S{U}^{st}(1,1)$ algebra which should satisfy

2.9 $${S}_{s}^{-}|lw\rangle =0\,{S}_{t}^{-}|lw\rangle =0$$

The lowest weight states, $|lw\rangle$ are a set of basis vectors as

2.10 $$|lw\rangle =|N,{k}_{s}=\frac{1}{2}\left({\nu }_{s}+\frac{1}{2}\right),{\mu }_{s}=\frac{1}{2}\left({n}_{s}+\frac{1}{2}\right),{k}_{t}=\frac{1}{2}\left({n}_{t}+\frac{3}{2}\right),{\mu }_{t}=\frac{1}{2}\left({n}_{t}+\frac{3}{2}\right)\rangle $$

In this relation, $N={v}_{t}+{\nu }_{s}$, ${n}_{t}={v}_{t}$, ${n}_{s}={\nu }_{s}=0$ or 1. Hence, we have

211 $$\begin{array}{rcl}{S}_{n}^{0}|l\omega \rangle  & = & \{{C}_{s}^{2n}{S}^{0}(s)+{C}_{t}^{2n}{S}^{0}(t)\}|lw\rangle \\  & = & \left\{{C}_{s}^{2n}\frac{1}{2}\left({n}_{s}+\frac{1}{2}\right)+{C}_{t}^{2n}\frac{1}{2}\left({n}_{t}+\frac{3}{2}\right)\right\}|l\omega \rangle \\  & = & {\Lambda }_{n}^{0}|l\omega \rangle \end{array}$$

The quantum phase transition between spherical and rotational nuclei is mainly driven by the nonzero nondiagonal part of the boson pairing operator. By employing the generators of $SU(1,1)$ algebra, the following Hamiltonian is constructed for the transitional region between $U\mathrm{(1)}\leftrightarrow SO\mathrm{(2)}$ limits

2.12 $$H=g{S}_{0}^{+}{S}_{0}^{-}+\alpha {S}_{1}^{0}$$

where and $\alpha$ are real parameters. It can be seen that Eq. (2.12) is equivalent to a Hamiltonian in $SO\mathrm{(2)}$ limit, when ${C}_{s}={C}_{t}$, and to a Hamiltonian in $U\mathrm{(1)}$ limit, when ${C}_{s}=0,{C}_{t}\ne 0$. Hence, the general ${C}_{s}\ne {C}_{t}\ne 0$ case corresponds with the $SO\mathrm{(2)}$ limit to the $U\mathrm{(1)}$ limit transitional region. In the following, ${C}_{t}$ is fixed to 1, and we allow ${C}_{s}$ to vary within the closed interval $[0,{C}_{t}]$.

To find the non-zero energy eigenstates with $k$-pairs, we exploit a Fourier Laurent expansion of the eigenstates of Eq. (2.12) in terms of unknown c-number parameters ${x}_{i}(i=1,2,\ldots ,k)$, so eigenvectors of the Hamiltonian for excitations can be written as:

213 $$|k;{\nu }^{s}{v}^{t}{n}_{\Delta }^{s}{n}_{\Delta }^{t};LM\rangle =\sum _{{n}_{i}\in z}{a}_{{n}_{1}}{a}_{{n}_{2}}\ldots {a}_{{n}_{k}}{x}_{1}^{{n}_{1}}{x}_{2}^{{n}_{2}}\ldots {x}_{k}^{{n}_{k}}{S}_{{n}_{1}}^{+}{S}_{{n}_{2}}^{+}\ldots {S}_{{n}_{k}}^{+}|lw\rangle $$

By using the commutation relations given by Eq. (2.2), it can be verified that all coefficients ${a}_{{n}_{1}}{a}_{{n}_{2}}\ldots {a}_{{n}_{k}}$ in Eq. (2.13) can be taken to be 1. The wave functions of Eq. (2.13) can be expressed simply as:

214 $$|k;{\nu }^{s}{v}^{t}{n}_{\Delta }^{s}{n}_{\Delta }^{t};LM\rangle =N{S}_{{n}_{1}}^{+}{S}_{{n}_{2}}^{+}\ldots {S}_{{n}_{k}}^{+}|lw\rangle $$

*N* is the normalization constant and,

2.15 $${S}_{{x}_{i}}^{+}=\frac{{C}_{s}}{1-{C}_{s}^{2}{x}_{i}}{S}^{+}(s)+\frac{{C}_{t}}{1-{C}_{t}^{2}{x}_{i}}{S}^{+}(t)$$

By using Eq. (2.14) and the commutation relations of Eq. (2.2) which leads to a set of Bethe Ansatz equations, the c-numbers ${x}_{i}$’s are determined by:

2.16 $$\frac{\alpha }{{x}_{i}}=\frac{g{C}_{s}^{2}({\nu }_{s}+\frac{1}{2})}{1-{C}_{s}^{2}{x}_{i}}+\frac{g{C}_{t}^{2}({v}_{t}+\frac{3}{2})}{1-{C}_{t}^{2}{x}_{i}}-\sum _{i\ne j}\frac{2g}{{x}_{i}-{x}_{j}}$$

These yield the eigenvalues $E(k)$ of Hamiltonian Eq. (2.12) in the form,

217 $$E={h}^{k}+\alpha {\Lambda }_{1}^{0},\,{h}^{(k)}=\mathop{\sum }\limits_{i=1}^{k}\frac{\alpha }{{x}_{i}},\,{\Lambda }_{1}^{0}={C}_{s}^{2n}\frac{1}{2}\left({n}_{s}+\frac{1}{2}\right)+{C}_{t}^{2n}\frac{1}{2}\left({n}_{t}+\frac{3}{2}\right)$$

The quantum number $k$ is related to the total number of bosons, $N=2k+{\nu }_{s}+{v}_{t}$. A useful and simple numerical algorithm for solving the BAE Eq. (2.16) and extraction of the constants in comparison with experimental energy spectra of considered molecules are based on using Matlab software which will be outlined simultaneously. To determine the roots of the BAE with specified values of and ${v}_{t}$, we solve Eq. (2.16) with definite values of $C$ and $\alpha$ for $i=1$ and then use the function “syms var” in Matlab to obtain all roots. To this aim, we have changed the variables as

218 $$C=\frac{{C}_{s}}{{C}_{t}}\,\preccurlyeq \,1,\,g=1,\,{y}_{i}={C}_{t}^{2}{x}_{i}$$

So, the new form of Eq. (2.16) would be

2.19 $$\frac{\alpha }{{y}_{i}}=\frac{{C}^{2}\left({\nu }_{s}+\frac{1}{2}\right)}{1-{C}^{2}{y}_{i}}+\frac{\left({\nu }_{t}+\frac{3}{2}\right)}{1-{y}_{i}}\sum _{i\ne j}\frac{2}{{y}_{i}-{y}_{j}}$$

We then repeat this procedure with different $C$ and $\alpha$ to minimize the root mean square deviation, σ, between the calculated energy spectra and experimental counterparts which explore the quality of extraction processes. The deviation is defined by the equality:

2.20 $$\sigma ={\left(\frac{1}{{N}_{tot}}|{E}_{\exp }(i)-{E}_{cal}(i){|}^{2}\right)}^{\frac{1}{2}}$$

${N}_{tot}$ is the number of energy levels that are included in the extraction processes. We have extracted the best set of Hamiltonian’s parameters via the available experimental data.

Similarly to $U\mathrm{(2)}$, this technique can be extended to the $U\mathrm{(4)}$ case. In the vibron model the rotations and vibrations are described in terms of four bosons: a scalar boson of positive parity and angular momentum $l=0$, denoted by ${s}^{\dagger }$, and the three components of a vector boson of negative parity and $l=1$, denoted by ${t}_{m}^{\dagger }$, $m=0,\pm \,1$. To this aim, we have used the same formalism to extend the $U\mathrm{(4)}$ calculation via $SU(1,1)$ Lie algebra. In the $U\mathrm{(4)}$ case, the Hamiltonian can be considered as

221 $$H=g{S}_{0}^{+}{S}_{0}^{-}+\alpha {S}_{1}^{0}+\beta {C}_{2}(O(3))$$

The eigenvalues of Eq. (2.21) can be expressed as

222 $${E}^{(k)}={h}^{(k)}+\alpha {\Lambda }_{1}^{0}+\beta (L(L+1))$$

In the study of molecular spectra, various approaches have been used. The Dunham expansion approach is very important. Rotational-vibrational molecular spectra are usually described in terms of the Dunham expansion

2.23 $$E(v,j)=\sum _{ik}{Y}_{ik}{\left(v+\frac{1}{2}\right)}^{i}{[j(j+1)]}^{k}$$

where $j$ is the angular momentum of the state, $v$ is the vibrational quantum number, and ${Y}_{ik}$ are the Dunham coefficients, which are fitted to experiment. The energy of rotational and vibrational levels of molecules can be investigated separately and summed. To do this, we use the characteristic values of the diatomic molecule. The investigation of the energy spectra in both vibrational and rotational states can be abbreviated as rovibrational (or ro-vibrational) transitions.

To find the band spectra in both rotating vibrators of the diatomic molecule, it would be convenient to use a Dunham expansion based on the quantization of the energy levels. The particular advantage of this method is that it gives a very good approximation to the actual energy levels by consideration of higher quantum effects. First of all, we calculate the energy spectra of the diatomic molecule by Dunham expansion then we reproduce these values by the algebraic approaches^27^.

The Dunham expansion to the same order in $v$ must be written as

2.24 $$\begin{array}{rcl}E(v,j) & = & \sum _{i=1}{Y}_{i0}{\left(v+\frac{1}{2}\right)}^{i}{[j(j+1)]}^{0}=\sum _{i=1}{Y}_{i0}{\left(v+\frac{1}{2}\right)}^{i}\\  & = & {Y}_{10}{\left(v+\frac{1}{2}\right)}^{1}+{Y}_{20}{\left(v+\frac{1}{2}\right)}^{2}+{Y}_{30}{\left(v+\frac{1}{2}\right)}^{3}+{Y}_{40}{\left(v+\frac{1}{2}\right)}^{4}+\ldots \end{array}$$

The ${Y}_{10}$ and ${Y}_{20}$ coefficients satisfy the relation $N=-\,\frac{{Y}_{10}}{{Y}_{20}}-2$, where $N$ is the total number of bosons. The maximum number of bound vibrational states is $N=2{v}_{m}$ or $2{v}_{m}+1$ for $N$ even or odd. Since all vibrational levels up to the dissociation energy are to be considered, the value needs to be determined accordingly. From the definition of the dissociation energy,

2.25 $${D}_{0}=E({v}_{max})-E(v=\mathrm{0)}$$

The value of $N$ determined from this scheme seems suitable for the whole vibrational spectrum.

### Transitional theory based on *q*-deformed algebra

In the preceding sections, the subalgebra chains of the model were reduced to equivalent chains of complementary subalgebras and the corresponding Hamiltonians were then written in terms of the Casimir operators of the new reduction chains. An evident possibility for $q$ deforming these Hamiltonians is to substitute the $SU(1,1)$ algebras by their $q$-deformed counterparts $SU(1,1)$^28,29^. The $q$-deformed Algebra has been explained in detail in refs. ^17,19,20^. For the sake of the $q$-deformation of the Hamiltonian in the $U(1)\leftrightarrow O(2)$ and $U(3)\leftrightarrow O(4)$ transitional region, the Casimir operators and generators should be written in $q$-deformed forms. The general $q$-deformed Hamiltonian can then be written as

2.26 $$H=g{S}_{0,q}^{+}{S}_{0,q}^{-}+\alpha {S}_{1,q}^{0}$$

2.27 $$H=g{S}_{0,q}^{+}{S}_{0,q}^{-}+\alpha {S}_{1,q}^{0}+\beta {C}_{2,q}(O(3))$$

where $q$ is the parameter quantum deformation and the parameter $q$ can be taken as real ($q={e}^{\tau }$ with $\tau$ real) or phase ($q={e}^{i\tau }$ with $\tau$ real). In the calculation, we take the $q$ number as phase, i.e., ${[x]}_{q}=\frac{sin\tau x}{sin\tau }$. The eigenvalue of Eqs. (2.26) and (2.27) can be expressed as

228 $$\begin{array}{rcl}E & = & {h}_{q}^{k}+\alpha {\Lambda }_{1,q}^{0}\\ E & = & {h}_{q}^{k}+\alpha {\Lambda }_{1,q}^{0}+\beta {[L]}_{q}{[L+1]}_{q}\\ {h}_{q}^{(k)} & = & \mathop{\sum }\limits_{i=1}^{k}\frac{\alpha }{{x}_{i}},\\ \frac{\alpha }{{x}_{i}} & = & \frac{g{C}_{s}^{2}{\left[\left({\nu }_{s}+\frac{1}{2}\right)\right]}_{q}}{1-{C}_{s}^{2}{x}_{i}}+\frac{g{C}_{t}^{2}{\left[\left({v}_{t}+\frac{3}{2}\right)\right]}_{q}}{1-{C}_{t}^{2}{x}_{i}}-\sum _{j\ne i}\frac{2}{{x}_{i}-{x}_{j}},\\ {\Lambda }_{1,q}^{0} & = & {C}_{s}^{2n}\frac{1}{2}\left({n}_{s}+\frac{1}{2}\right)+{C}_{t}^{2n}\frac{1}{2}\left({n}_{t}+\frac{3}{2}\right)={\Lambda }_{1}^{0}\end{array}$$

## Numerical result

In this section, we investigate the extent to which the Hamiltonians can describe experimental spectra. The cases discussed above are interesting because they provide analytic expressions for the properties of the system that can be easily compared to the experiment. In our considered framework, we have compared the predictions of the transitional Hamiltonian for energy spectra with their experimental counterparts. On the other hand, predictions of our model for the control parameter, $C$, one may conclude that our considered control parameter has the same role as the mixing parameter of other investigations which explains the combination of vibration and rotation or rovibrational configurations. This means that for these numbers of levels in this energy region, the affine $SU(1,1)$ approach can be regarded as the more exact method for describing the rovibrational energy levels of the considered molecules in the transitional.

In this work, we consider the homonuclear diatomic molecule ${H}_{2}$ and ${N}_{2}$ in its ${x}^{1}{\Sigma }_{g}^{+}$ state for our purpose. Dunham coefficients (${Y}_{i0}$) for ${H}_{2}$ and ${N}_{2}$ molecules were taken from refs. ^30–32^. Since in this state of ${H}_{2}$ and ${N}_{2}$ it is experimentally known that ${v}_{max}=10$ and ${v}_{max}=49$, respectively. We consider $N=21$ ($N$ can be either 20 or 21) and $N=99$ ($N$ can be either 98 or 99) for ${H}_{2}$ and ${N}_{2}$, respectively. These two type of homonuclear molecules fitted by using $U(1)\leftrightarrow O(2)$, $U(3)\leftrightarrow O(4)$ and ${U}_{q}(3)\leftrightarrow {O}_{q}(4)$ theory. All experimental vibrational levels including those not observed up to now are obtained by using the Dunham expansion formula with a set of vibrational spectroscopy constants confirmed by experiment, which is denoted as .

The fitting results, parameters, and errors in fits are given in Tables 1 and 2. The results show that the fit of the ${U}_{q}(3)\leftrightarrow {O}_{q}(4)$ theory is better than of the $U(1)\leftrightarrow O(2)$ and $U(3)\leftrightarrow O(4)$ theories. The results of $U(3)\leftrightarrow O(4)$ suggest more exact outcomes, i.e. minimum $\sigma$ values, in comparison with the experimental data and also with the $U(1)\leftrightarrow O(2)$ predictions.

**Table 1:** Comparison of calculated energy levels and Dunham expansion spectra^30,32^ of the grand state, ${x}^{1}{\Sigma }_{g}^{+}$, of the *H*_2_ molecule.

*E*_*Dunham*_	*v*_*t*_	*ν*_*s*_	*k*	*E*_*U*(1)↔*O*(2)_	*E*_*U*(3)↔*O*(4)_	${{\boldsymbol{E}}}_{{{\boldsymbol{U}}}_{{\boldsymbol{q}}}({\bf{3}})\leftrightarrow {{\boldsymbol{O}}}_{{\boldsymbol{q}}}({\bf{4}})}$
2170.7	0	1	10	5527.8	4001.2	2150.5
6332.3	1	0	10	8832.2	5957.5	6341.2
10261.8	2	1	9	12388.5	11077.1	10369.17
1394.8	3	0	9	12997.2	14475.9	13826.52
17453.03	4	1	8	15325.2	18418.8	17239.9
20733.8	5	0	8	17332.9	21041.8	20801.2
23815.5	6	1	7	20268.9	24963.4	23099.7
26705.04	7	0	7	24256.6	28001.3	26383.1
29408.43	8	1	6	34217.4	28656.5	28571.4
31929.3	9	0	6	35428.7	30897.5	31863.2
34268.22	10	1	5	35605.97	34772.8	33206.75
**parameters**						
_*σ*_				2935.8	968.94	479.47
_*α*_				2170.5	1985.8	1289.3
_*β*_				—	57.9	101.7
_*τ*_				—	—	0.031
_*C*_				0.78	0.89	0.89

The parameters of the fits are shown in the lower part of the table. All parameters are given in cm^−1^. In all cases, *N* has been fixed to 21 and *τ* (the deformation of the algebra) was used as free parameters. *σ* is the quality indicator defined in Eq. (2.20).

**Table 2:** Comparison of calculated energy levels and Dunham expansion spectra^30^ of the grand state, ${x}^{1}{\Sigma }_{g}^{+}$, of the *N*_2_ molecule.

*E*_*Dunham*_	*v*_*t*_	*ν*_*s*_	*k*	*E*_*U*(1)↔*O*(2)_	*E*_*U*(3)↔*O*(4)_	${{\boldsymbol{E}}}_{{{\boldsymbol{U}}}_{{\boldsymbol{q}}}({\bf{3}})\leftrightarrow {{\boldsymbol{O}}}_{{\boldsymbol{q}}}({\bf{4}})}$
1175.7	0	1	49	1236.1	1197.31	1180.9
3505.6	1	0	49	2766.8	2956.8	3622.3
5806.9	2	1	48	4590.2	5007.7	5745.9
8079.4	3	0	48	4617.9	6124.7	7884.3
10323.2	4	1	47	9711.3	8956.4	9991.2
12538.24	5	0	47	11451.1	11046.2	12404.5
14724.5	6	1	46	12001.8	13518.7	14908.1
16881.8	7	0	46	15101.2	16118.9	16821.8
19010.3	8	1	45	16987.1	18414.1	18997.3
21109.8	9	0	45	19934.5	20948.3	21053.4
23180.2	10	1	44	22656.1	22874.5	23006.2
25221.6	11	0	44	24010.1	24434.2	25120.7
27233.7	12	1	43	25361.4	26889.7	27114.9
29216.6	13	0	43	27273.2	28544.3	29006.1
31170.14	14	1	42	29801.7	31331.0	31231.5
33094.2	15	0	42	31463.3	32713.8	33152.3
34988.7	16	1	41	32762.2	33549.5	35018.7
36853.6	17	0	41	35256.7	36014.7	36915.9
38688.7	18	1	40	36221.6	37817.2	38840.7
40493.9	19	0	40	39111.1	39111.8	40660.2
42269.2	20	1	39	41568.7	41988.2	42347.7
44014.24	21	0	39	43004.0	43746.6	44170.7
45729.05	22	1	38	46661.2	44894.2	46010.6
47413.4	23	0	38	47300.2	47152.9	47345.4
49067.3	24	1	37	48001.4	48358.9	49486.8
50690.4	25	0	37	48796.1	49134.6	50899.3
52282.6	26	1	36	51397.3	51860.5	52432.1
53843.9	27	0	36	52258.3	52409.8	54056.7
55373.9	28	1	35	54310.5	54243.9	55514.4
56872.6	29	0	35	56560.7	55817.4	5682.4
58339.7	30	1	34	57247.1	57374.2	57722.5
59775.03	31	0	34	58522.1	58846.4	59953.4
61178.46	32	1	33	59754.4	60697.4	61129.3
62549.7	33	0	33	61397.01	61925.1	62772.8
63888.7	34	1	32	62323.5	63081.2	63707.1
65195.13	35	0	32	64879.6	64984.6	65289.6
66468.8	36	1	31	65785.8	65358.9	66735.4
67709.5	37	0	31	65896.7	67114.5	67419.2
68916.93	38	1	30	66642.1	67396	68728.5
70090.96	39	0	30	66923.4	69022.3	70104.2
71231.3	40	1	29	68013.7	70236.7	71435.5
72337.7	41	0	29	68999.8	71019.9	72587.7
73410.0	42	1	28	71641.3	72176.8	73243.1
74447.8	43	0	28	72343.9	73856.6	74438.9
75450.98	44	1	27	74271.2	74249.8	75432.4
76419.2	45	0	27	75101.7	75717.1	76529.9
77352.13	46	1	26	75954.3	76343.2	77443.2
78249.6	47	0	26	76897.0	77470.7	78335.9
79111.25	48	1	25	77176.1	78152.8	78915.9
79936.8	49	0	25	77531.4	78895.3	81591.2
**parameters**						
_*σ*_				1676.1	948.14	305.98
_*α*_				1473	921.2	1005.2
_*β*_				—	83.5	73.5
_*τ*_				—	—	0.036
_*C*_				0.83	0.97	0.97

The parameters of the fits are shown in the lower part of the table. All parameters are given in cm^−1^. In all cases, *N* has been fixed to 99 and *τ* (the deformation of the algebra) was used as free parameters. *σ* is the quality indicator defined in Eq. (2.20).

The spectra of diatomic molecules in the framework of the $U\mathrm{(4)}$ and model were considered in refs. ^14,30–32^. So, it must be useful and worthwhile to compare the present method and results in the method and results of these papers. The paper by Xin & Feng^33^ investigated the transitional description of diatomic molecules in the $U\mathrm{(4)}$ vibron model. Our result for ${N}_{2}$ is more precise than their result. In ref. ^14^, Bonatsos *et al*. studied quantum algebraic description of vibrational molecular spectra in ${H}_{2}$. Comparing results in $U(3)\leftrightarrow O(4)$ and ${U}_{q}(3)\leftrightarrow {O}_{q}(4)$ with ref. ^14^, It is observed an agreement between our results with the previously reported results.

We have found, the $U(3)\leftrightarrow O(4)$ formalism increases phase parameter $C$ weight in the calculation. Besides, it should also be noticed that phase parameter $C$ plays a significant role in these theories. The phase parameters for ${H}_{2}$ and ${N}_{2}$ in this analyse are in the range of 0.78~0.89 and 0.83~0.97. Thus, we conclude that ${H}_{2}$ and ${N}_{2}$ diatomic molecules are mixing both vibrating and rotating structures.

## Conclusion

Using the Lie algebraic method based on quantum deformed and nondeformed, we reported in these methods for a diatomic molecule. We have presented here an algebraic approach to molecular rotation-vibration spectra. In this work, we have confined ourselves to the study of diatomic molecules, in order to introduce phase transition based on deformed and nondeformed employed in $U\mathrm{(2)}$ and $U\mathrm{(4)}$ limits. The approach is general enough in that it can describe both rigid and nonrigid molecules. The results indicate that the energy spectra can be reproduced quite well. The quantum deformation technique enables us to input all high-order terms of a certain type and only add a few parameters to the Hamiltonian, which can be regarded as a possible extension.
